# An instrument for broadened risk assessment in antenatal health care including non-medical issues

**DOI:** 10.5334/ijic.1512

**Published:** 2015-03-06

**Authors:** Amber A. Vos, Mieke J. van Veen, Erwin Birnie, Semiha Denktaş, Eric A.P. Steegers, Gouke J. Bonsel

**Affiliations:** Department of Obstetrics and Gynaecology, Division of Obstetrics & Prenatal Medicine, University Medical Centre Rotterdam, Rotterdam, The Netherlands; University of Applied Science, Centre of Expertise Innovations in Care, Rotterdam, The Netherlands; Institute of Health Policy and Management, Erasmus University, Rotterdam, The Netherlands; Department of Obstetrics and Gynaecology, Division of Obstetrics & Prenatal Medicine, University Medical Centre Rotterdam, Rotterdam, The Netherlands; Department of Social & Behavioural Sciences, Erasmus University College, Rotterdam, The Netherlands; Department of Obstetrics and Gynaecology, Division of Obstetrics & Prenatal Medicine, Erasmus MC, University Medical Centre Rotterdam, Rotterdam, The Netherlands; Department of Public Health, University Medical Centre Rotterdam, Rotterdam, The Netherlands; Department of Obstetrics and Gynaecology, Division of Obstetrics & Prenatal Medicine, University Medical Centre Rotterdam, Rotterdam, The Netherlands

**Keywords:** non-invasive risk screening, risk assessment, integrated care, care pathway, perinatal health, pregnancy

## Abstract

**Introduction:**

Growing evidence on the risk contributing role of non-medical factors on pregnancy outcomes urged for a new approach in early antenatal risk selection. The evidence invites to more integration, in particular between the clinical working area and the public health domain. We developed a non-invasive, standardized instrument for comprehensive antenatal risk assessment. The current study presents the application-oriented development of a risk screening instrument for early antenatal detection of risk factors and tailored prevention in an integrated care setting.

**Methods:**

A review of published instruments complemented with evidence from cohort studies. Selection and standardization of risk factors associated with small for gestational age, preterm birth, congenital anomalies and perinatal mortality. Risk factors were weighted to obtain a cumulative risk score. Responses were then connected to corresponding care pathways. A cumulative risk threshold was defined, which can be adapted to the population and the availability of preventive facilities. A score above the threshold implies multidisciplinary consultation between caregivers.

**Results:**

The resulting digital score card consisted of 70 items, subdivided into four non-medical and two medical domains. Weighing of risk factors was based on existing evidence. Pilot-evidence from a cohort of 218 pregnancies in a multi-practice urban setting showed a cut-off of 16 points would imply 20% of all pregnant women to be assessed in a multidisciplinary setting. A total of 28 care pathways were defined.

**Conclusion:**

The resulting score card is a universal risk screening instrument which incorporates recent evidence on non-medical risk factors for adverse pregnancy outcomes and enables systematic risk management in an integrated antenatal health care setting.

## Introduction

Perinatal health is a topic of growing international concern. Two subsequent reports on perinatal health concluded that within European countries impressive inequalities exist in perinatal outcomes [1,2]. Evidence from large cohort and registry studies clearly demonstrated an equally high impact on perinatal outcomes of non-medical risk factors (e.g. social of lifestyle) compared to medical and obstetrical risk factors [3,4].

These etiological studies also showed that the cumulative presence of a set of heterogeneous risk factors of moderate importance-rather than the presence of a single large risk-underlies most adverse outcomes. This so-called risk accumulation is especially observed in deprived geographical areas characterized by overrepresentation of women with low socio-economic status, single parenthood, migrant status and numerous associated risks (medical, non-medical or both) [5–7].

More than 85% of all cases of perinatal mortality are associated with only four adverse perinatal outcomes, either single or combined. These so-called Big 4 outcomes include congenital disorders, small for gestational age, preterm birth and/or suboptimal start at birth (low Apgar score) [8]. Big 4 outcomes are in general closely related to medical and non-medical risk factors. They represent the link between increased perinatal mortality rate and their associated risk factors. Many of these risk factors are already present at onset of pregnancy.

This new evidence on risk pathways invites to more interdisciplinary collaboration, in particular between the clinical working area and the public health domain when non-medical plays an essential role (horizontal integration). In this respect, a comprehensive risk model with associated integrated care delivery could underlie the antenatal health care system [9]. Integrated care aims to ‘deliver services across providers with minimal duplication and disruption, and with high-quality outcomes and patient experience’ [10]. When translated to antenatal health care with a comprehensive risk model, this implies a shared risk, shared management and shared care provision concept. This comprehensive risk models pays equal attention to medical and non-medical risk factors, both in the assessment of risk levels and the therapeutic and preventive measures.

The relatively unfavourable position of the Netherlands regarding perinatal mortality [1], in particular in urban areas [11], resulted in the development of shared care as the model for antenatal health care [9]. This comprehensive model acknowledges the relevance of accumulation of heterogeneous risks by introducing universal, broad antenatal risk assessment including medical and non-medical risk factors. Broad risk assessment is followed by multidisciplinary responsibility to reduce high risk cases [8,12].

However, risk assessment lacked a comprehensive tool as the available routine antenatal screening instruments focus mainly on medical factors [13–17] and do not consider risk accumulation as mechanism. The few instruments available are rarely used beyond the research setting [16] and not embedded in integrated care settings.

In response to the need for a comprehensive tool which connects medical and non-medical risks to integrated pathways, we developed a non-invasive, standardized score card for routine use in antenatal health care. The ‘Ready for a baby’ programme, a prior antenatal health care programme in the city of Rotterdam [5], provided the framework to develop and pilot this instrument in the antenatal health care setting [18]. The main purpose of this universally applicable score card is early identification of women with an increased risk for common adverse pregnancy outcomes, notably small for gestational age and prematurity. It offers tailored preventive and curative options for both conventionally detected risk factors (e.g. cocaine use) and for non-medical risk factors (e.g. domestic violence or financial debts). Following the current ‘developmental origin hypothesis of adult disease’, it is in the interest of the future health and development of the child to maximize efforts to reduce avoidable (non-)medical risk factors at the earliest possible stage [19].

The aim of this study is to present the further application-oriented development of this risk screening instrument for early antenatal detection of risk factors and tailored prevention in an integrated care setting. This paper presents an overview of published risk assessment instruments, the methodological and clinical considerations of associated with the development of our instrument, and the application-oriented step from a score card risk profile to an integrated risk modifying approach of detected risk factors with the use of so-called care pathways in a multidisciplinary setting.

## Background

In Box 1, we provide the conceptual framework which illustrates the interaction of non-medical and medical risk determinants and their influence on adverse perinatal outcome. Not all underlying mechanisms are revealed. Frequently factors are bidirectional-related, as have been pointed out by researchers in the area of deprivation research [11]. Regarding non-medical risk factors, socio-economic status and neighbourhood deprivation are most consistently related to adverse perinatal outcome. Socio-economic status can induce adverse perinatal outcome though multiple pathways, most importantly through low education and low income levels [20]. Previous studies showed that decreased wealth and poor housing increase physiological stress [21]. Low income levels and deprivation are also associated with poor housing, nutritional deficiencies and impaired health care access [7,11,22]. However, to date it is unknown to what extent the effect of deprivation goes beyond the effect of poor individual level of socio-economic status of citizens in deprived neighbourhoods [23,24].

Regarding medical risk factors, several diseases or disease-related pharmaceuticals directly affect perinatal outcome. Effects are induced by the disease itself (e.g. increased risk for growth restriction in some auto-immune diseases) or can be more indirectly related (e.g. the increased risk for still birth in poorly regulated diabetes mellitus). Most obstetric adverse outcomes show an increased recurrence rate in subsequent pregnancies.

Lastly, non-medical risk factors also act indirectly through their general adverse health effects: low socio-economic status is associated with a higher prevalence and poor prognosis for most common diseases and may decrease life expectancy up to 10 years in developed countries [25,26].

## Methods

### General

Our risk assessment instrument, the so-called Rotterdam Reproductive Risk Reduction (R4U) score card, was designed as a professional based risk assessment instrument applicable during the first antenatal visit. The first antenatal visit takes normally takes place before the 12th week of gestation. It focused on risks associated with the occurrence of Big 4 outcomes at birth and with perinatal mortality. Risks may have a medical or a non-medical background [18]. The R4U scorecard can be used as stand-alone or combined with other tests. The latter means that the scorecard could also be combined with other (pre-existing) screening tests if more extensive screening on specific risk factors is necessary (triage purpose). The final aim of the scorecard was to link responses to clinical management protocols.

### Risk factors selection for the score card

The initial selection of risk factors for the first version included a review of existing risk assessment instruments and a selected literature research for specific domains and items [18]. In view of the pilot success in terms of feasibility and acceptability of the pilot score card, the present study repeated the literature underpinning through a formal design and adding some additional features of interest. Attention was restricted to published instruments from western countries from 1990 onwards. Score cards published until 1990 were described by other studies and therefore excluded for this purpose [13,15–17]. We performed a broad electronic literature search 3rd December 2012 in Medline, Embase and Web of Science from inception to December 2012. A search strategy was developed based on antenatal risk screening and its synonyms such as ‘antenatal risk assessment’, ‘antenatal risk screening’, ‘scorecard’ which were also combined with ‘pregnancy’, ‘prenatal’ and ‘obstetric’. Although we hand searched reference lists from main articles and relevant reviews for additional eligible studies, the synonyms were numerous. No language restrictions were applied. The remaining risk assessment instruments related to antenatal health care were included.

Each published instrument was structurally reviewed along the following topics: predicted outcome (e.g. low birth weight, or adverse outcome as whole), timing of screening (e.g. first antenatal visit, or selected gestational trimester), population (e.g. whole population, low income women), registration (item selection, e.g. social items, obstetrical items), scoring (summation of items, weighing of items; use of cut-offs), practical disadvantages, validation, discriminative power and current practical use. At the second stage, we complemented this review with detailed epidemiological information (prevalence and risk estimates) from well-documented large birth cohort studies which have published risk factor analysis for various birth outcomes [27–30]. At a third stage we completed the candidate list with risk factors suggested by published (inter)national guidelines on prenatal assessment [31,32].

### Design of the score card

#### Item selection

Risk factors were selected if unequivocal evidence pointed to an association with small for gestational age, preterm birth, and congenital anomalies (‘Big 3’), and perinatal mortality. While we initially aimed to detect pregnancies prone to a delivery with a low Apgar score, we excluded this aim as insufficient evidence exists on its suitability to predict in such an early stage. Initial selection of risk factors incorporated in the R4U score card took place in five expert meetings within the project group [18]. All selected risk factor questions (‘items’) were standardized. To increase uniformity, we defined a so-called ‘script’ text for each separate item as a literal text to list the question. It was printed at the back of paper and pencil forms or could be popped up in the digital form underlying the present study. It was thought that the script may facilitate questioning of sensitive questions (e.g. domestic violence) and decreases error in questions with known intra-professional variation (e.g. miscarriage, living in a deprived neighbourhood).

The standard format of question and closed response was derived from the Woman, Infants and Children prenatal risk factor score card. Response was dichotomous only. The ‘yes’ was an indicator for the presence of the risk factor with a known relative risk of adverse perinatal outcomes. The resulting list of candidate items was first piloted for feasibility and reliability [18].

#### Connection of risk profile to care pathways

The present study added a connection between risk profile as established through the R4U scorecard and a tailored care pathway. To this purpose, each item was categorized into four groups according to their instrumental use:

- Single risk: such a risk factor that is directly linked to Big 3 and/or perinatal mortality and justifies intervention independent from other considerations (e.g. drug use).
- Additive risk: such a risk factor that is associated with Big 3 and/or perinatal mortality (both medical and non-medical risk factors, irrespective of the avoid ability of the risk factor) and has been reported to contribute to adverse outcome as cumulative risk (e.g. lack of social support or being unemployed).
- Conditional risk: such a risk factor that is known to be relevant for Big 3 or perinatal mortality, but being a risk factor dependents on more information, e.g. being unemployed is a risk factor if family income depends on maternal employment, but may be of minor importance if a partner provides sufficient income.
- Instrumental risk: from the Woman, Infants and Children prenatal risk factor score card [33], we adopted the inclusion of some risk factors which are not associated with adverse perinatal outcome, but which could strongly affect the provision of perinatal care. Detection of these risk factors creates awareness by the care professional, e.g. Jehovah's Witness (blood transfusion), illiteracy (health promotion and instruction materials) and ethnic background (the use of interpreter services; the taking into account for specific cultural habits and expectations).

Occasionally risk factors belong to more than one category. A single risk of, e.g., preterm birth may also act as additive risk for other adverse outcomes.

In the development, we also introduced ‘weighing’ and the cumulative risk score. To obtain a cumulative risk score from an individual profile of positive risk items, weights have to be assigned to each abnormal'yes' category. A cumulative risk score above a predefined cut-off point would imply a follow-up action, including multidisciplinary consultation between perinatal professionals and other health care professionals, such as paediatricians or social workers. It offers the opportunity to customize antenatal policy to the individual woman's need. Such a cut-off may also be locally adapted to accommodate the availability of facilities. The present weights were obtained from published odds ratios and relative risks in large, representative birth cohort studies, meta-analysis and in an occasional case–control study (source data available upon request). We expressed weights in points, depending on the odds ratios/relative risks of a risk factor: risk factors consistently associated with odds ratios/relative risks smaller than two were assigned 1 point, higher than two or related to perinatal mortality were assigned 2 points, and for risk factors associated with odds ratios/relative risks higher than four, 3 points were assigned. For a few items, assignment of a weight was primarily based on expert opinion. Occasionally publication delay underrated current practice: e.g. previous stillbirth or previous Small for gestational age justified a higher weight than papers so far suggested. These items were expert opinion prevailed, for example, in case of a high relapse rate (e.g. preconceptional use of drugs). The remaining items, for which there was currently no evidence available, received 0 points.

The use of both paper and pencil (A4-format) and digital score card was intended. The first version employed a paper and pencil version following the two column lay-out of the Woman, Infants and Children prenatal risk factor score card [33]. The digital form used an open source software system to present a digital questionnaire to the health care professional.

### Service responses to the score card

As mentioned previously, responses from the R4U score card (the risk profile) were connected to corresponding care pathways. Care pathways were included to address the management of (non-)medical risk factors [9]. We developed 28 templates of care pathways for all risk factors (single and cumulative) incorporated in the R4U score card.

These care pathways not only support conventional medical and obstetrical risk factors but also incorporate unconventional, non-medical risk factors. Each care pathway consists of a defined set of measures a health care professional could take to meet the specific need of the pregnant women. Predesigned templates should be adapted to the local settings to fulfil local needs. The details and service response will be described in detail in the section ‘Practical Experiences’.

### Pilot study

The resulting R4U risk score card was piloted in several hospitals and midwifery practices in Rotterdam. Data collection of this pilot study on feasibility and reliability took place from 2010 until 2011 [18].

In the ‘Result’ section below, we provided the results of the developmental steps of the R4U score card, including the risk factor selection and categorization, the summary score procedure, and the application-oriented extension from screening to tailored care provision through care pathways. The results include the subsequent modifications derived from the first pilot study. To illustrate the potential service impact of the R4U score card, we provided an illustration of the summary score and the application of a threshold was with the use of real data from a first pilot in practice.

Approval was obtained from the Institutional Review Boards of the Erasmus Medical Centre before the study began and decided that written informed consent was not necessary.

## Results

Table 1 presents a review of eight predictive instruments for routine use in antenatal care published from 1990 onwards [17,33–39]. Five out of eight instruments focused on more than one predictive outcome; for one the predicted outcome is unknown. None of the instruments took congenital anomalies into account. Six instruments can be applied to identify risks at the first antenatal visit, and all except one are used for an unselected group of pregnant women, rather than a specific group. The instruments often specialized on either medical or non-medical factors, rather than a combination; the number of items included ranges from 4 to 45. In seven instruments a validation test of the claimed predictive power had taken place, among these are only two instruments that are externally validated. The positive predictive value was available for four instruments, ranging from 1.4% to 33%. As far as we know, four instruments were currently in use.

In Table 2, we show the contents of the R4U score card in terms of domains, items, item relevance and weighing factors. The final R4U score card included 70 items categorized into six domains, namely social status (*n* = 14), ethnicity (*n* = 3), reproductive factors (*n* = 8), lifestyle (*n* = 14), medical history (*n* = 14) and obstetric history (*n* = 17). The assignment of item categories (1–4) in the column ‘item relevance’ was based on consensus by the project group. Items were grouped according to subject for ease of history taking. In the paper and pencil version, the four non-medical domains are listed on the left side (*n* = 39 items), and the two medical domains on the right side (*n* = 31 items). The digital version presents the items one by one. As explained previously, for some items the assigned weight was expert opinion based: small for gestational age, previous still birth, short interpregnancy interval, body mass index >35 kg/m^2^, living in a deprived neighbourhood, preconceptional smoking and preconceptional illicit drug use. For other items, risk estimates were dependent on nature of the disease which varies across women: prescribed drugs during pregnancy, type of psychiatric disorder, recurrence rate of congenital anomalies, major congenital anomalies in first degree relative and positive booking bloods. In case of item category ‘4’ zero points were allocated for the summary score.

**Figure 1.**
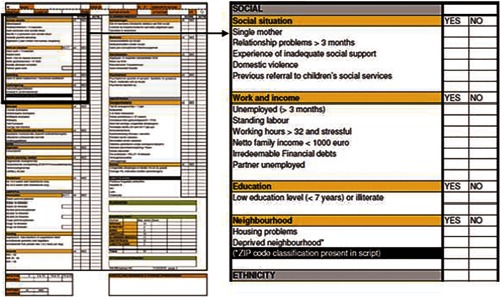
Elaborated example of one domain in R4U

Figure 1 shows an excerpt of the R4U score card in its original paper and pencil form (social domain). A paper and pencil version cannot apply automated skipping of irrelevant items nor the automated summation of weights. The whole R4U score card is added as Supplementary File (Supplement 1).

**Figure 2.**
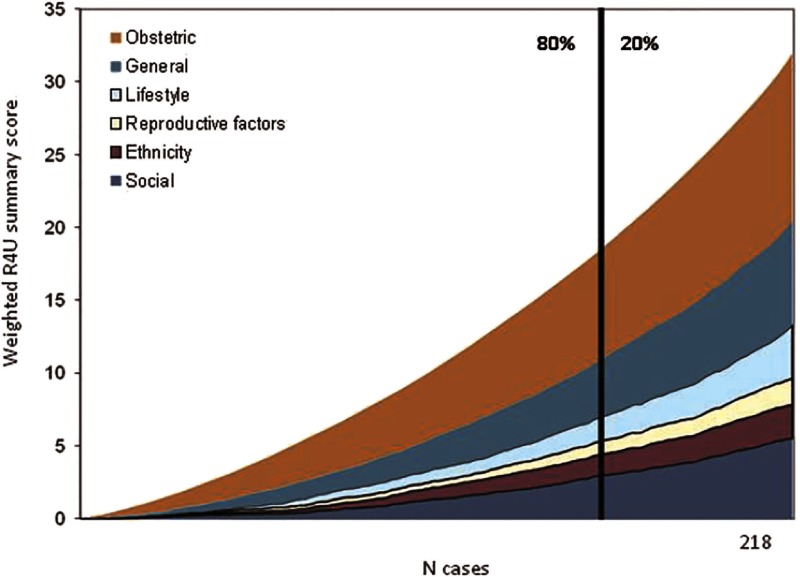
Cumulative weighted R4U summary scores for all domains separately for the first 218 cases of the pilot study

Three midwifery practices and two hospitals used the paper and pencil version R4U score card during their first antenatal visit [18]. The pilot version items were closely related to the current version as items rarely need adaptation. From the first 218 pregnancies, we derived the weighted summary scores as presented in Figure 2. Pregnancies were sorted on ascending summary scores. The figure illustrates that each domain contributes from the beginning to our summary scores, and at any risk level all domains contribute to the risk load. The cut-off of 16 points indicated an advice for multidisciplinary consultation in 20% of all pregnant women.

## Practical experience

The R4U score card was proposed to facilitate improved coordination of antenatal care through systematic and uniform risk screening for medical and non-medical risk factors [18]. Via 28 predefined care pathways it contributed to unequivocal division of tasks and responsibilities for non-medical risk factors. While we here have described the use of the R4U as instrument for triage, optimal profit from the risk information arises if care pathways were connected to the individual needs of pregnant woman [9]. Care pathways were used to modify these risk factors, and as these pathways are explicit as to which caregiver will be responsible, the efficiency and accountability are enhanced. Together with local health care professionals in perinatal care, municipal services, community health services and other services, these pathways were projected on the local setting in organized meetings. This means that the availability of local facilities and insurance agreements were taken into consideration.

In addition, a risk score above the predefined cut-off point implied follow-up action. This follow-up action included multidisciplinary consultation between obstetric caregivers and non-obstetric caregivers, prioritization of risk factors, and feedback in subsequent meetings. For the client, an effect of this approach was the prevention of doubling of history taking by different professionals at different stages. The standardized format facilitated risk communication across disciplines.

The introduction of organized meetings to customize care pathways induced a change in the mutual professional relationship. Initially obstetric caregivers and non-medical caregivers functioned strictly separate, each covering their ‘own’ part of the case. The organized meetings induced mutual respect and much more awareness among both professionals on the impact and effect of non-medical risk factors. It realized the necessity to address these non-medical risk factors to improve perinatal outcome.

Early detection and active involvement of ‘non-medical caregivers’ through care pathways provided the opportunity to monitor actively from on the onset of pregnancy. This was not the case in the conventional scenario in which non-medical risk factors were passively noticed. Note that this approach might induce a shift in workload to the early antenatal phase, including more registration for monitoring.

The R4U scorecard enables a considerable change in early antenatal health care as illustrated in Box 2. Here we present the impact on the service response of the comprehensive R4U-based approach versus the conventional approach in a representative case example.

## Discussion

The resulting R4U score card is proposed as a universal risk screening instrument. It incorporates recent evidence on non-medical risk factors for adverse pregnancy outcomes and could facilitate an integrated antenatal health care setting. Unique is the close connection between risk profile and care pathways focusing on non-medical factors. The intended users are obstetric professionals, such as midwives, obstetricians, obstetrical nurses or general practitioners, and it can be applied to all pregnancy populations. An integrated setting (including psychosocial and public health workers) is preferable for optimal use, as the resulting care pathways require medical and non-medical expertise. The R4U score card is a method for integrated risk management, which facilitates coordination of antenatal health care through uniform risk screening, and a clear division of tasks when high risk cases are treated through predefined care pathways. The score card enables and supports a shared care provision model [9].

Through its concept and standardized assessment, it bridges the gap between the clinical working area and the public health domain (‘minimal duplication and disruption’). By the inclusion of risk factors that were directly associated with adverse perinatal outcome and uniform definition of risks by all professionals involved, it enhances the ultimate aim to reduce adverse perinatal outcome (‘high-quality outcomes’). Care pathways add to efficiency and task division in multidisciplinary settings. This all facilitates effective and efficient management of women at risk, without ambiguity who is in charge [9,10].

Risk scoring in apparently asymptomatic persons for triage purposes is also known from other specialties such as the Framingham Coronary Heart Disease Prediction Scores for predicting risk of clinical coronary heart disease events [40]. In obstetrics, risk scoring is at this moment rarely routinely applied-and if so-only for obstetrical risk factors. We believe that the emerging knowledge on the predictive role of both medical and non-medical risk factors and risk accumulation justifies the introduction of a formal ‘evidence-based’ screening tool. Inspired by the Women, Infants and Children antenatal score card, we aimed to develop an extended version, anticipating on multiple risk factors which commonly determine care and support.

We are aware of the fact that the performed search on previous instruments was still an exploration of the rapidly expanding literature. There may still be methods that we have overlooked that could be a valuable addition. In addition, we omitted some instruments without apparent scientific evaluation. However, since we have also checked professional guidelines at the international level, we believe the probability is low that we overlooked a checklist with important application.

Although the current version of the score card covers 70 common items, we are well aware on the potential presence of rare risks. A score card cannot replace professional responsibility for careful history taking, as is also true with the use of for example cardiologic prediction scores and surgical checklists [41]. Over time, items can be added or deleted or adapted to local circumstances.

For the implementation of such a new approach in antenatal risk assessment, a number of conditions need to be fulfilled. Most importantly, professionals from obstetric care, social welfare, psychiatric services and community health services should agree on the comprehensive risk concept as a base to collaborate. Conflicting financial incentives and existing inter-professional communication barriers may represent a challenge for the implementation of this innovative screening plus intervention method [9]. Health care professionals may need training to question and encounter the new non-medical risk factors, and the time schedule of the first antenatal visit may require adaptation. Health insurance reimbursement schemes might need revision in order to enable this. This is also true for the non-medical preventive and curative measures following the care pathways. However, the administration of the R4U score card is not the time consuming part of antenatal health care, rather the professional effort to guide the detected high women being compliant to the proposed care pathways.

Future research is necessary to investigate the performance of the R4U score card under routine conditions and to measure the extent of integrated care it invokes [10]. The feasibility, inter-intra observer variability and predictive value are investigated, and it seems that the R4U risk score card is a feasible and reliable instrument [18]. A nationwide randomized controlled trial recently started to establish the effectiveness of early systematic antenatal risk detection with the R4U on pregnancy outcomes [42]. Over time the effectiveness and efficiency of this comprehensive risk assessment should be clarified-currently little is known on the cost-effectiveness of routine antenatal screening.

## Conclusion

The present study describes the development of the R4U score card, a non-invasive, standardized instrument for routine early antenatal risk screening which covers both medical and non-medical risk factors. The R4U score card is designed as a universal, non-invasive risk screening instrument incorporating recent evidence on non-medical risk factors for adverse birth outcomes and their treatment to facilitate integrated antenatal health care.

## Figures and Tables

**Figure:**
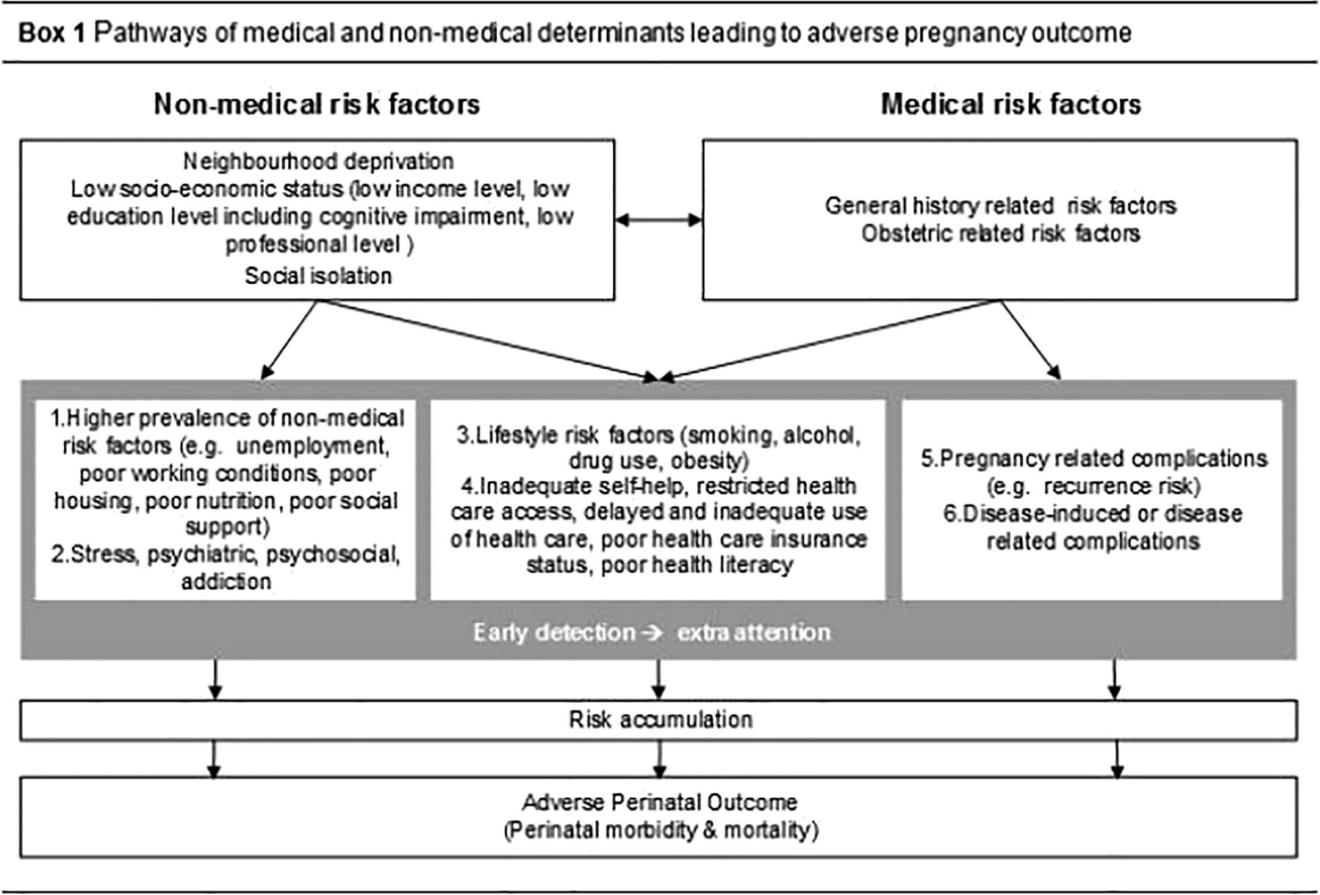


**Figure:**
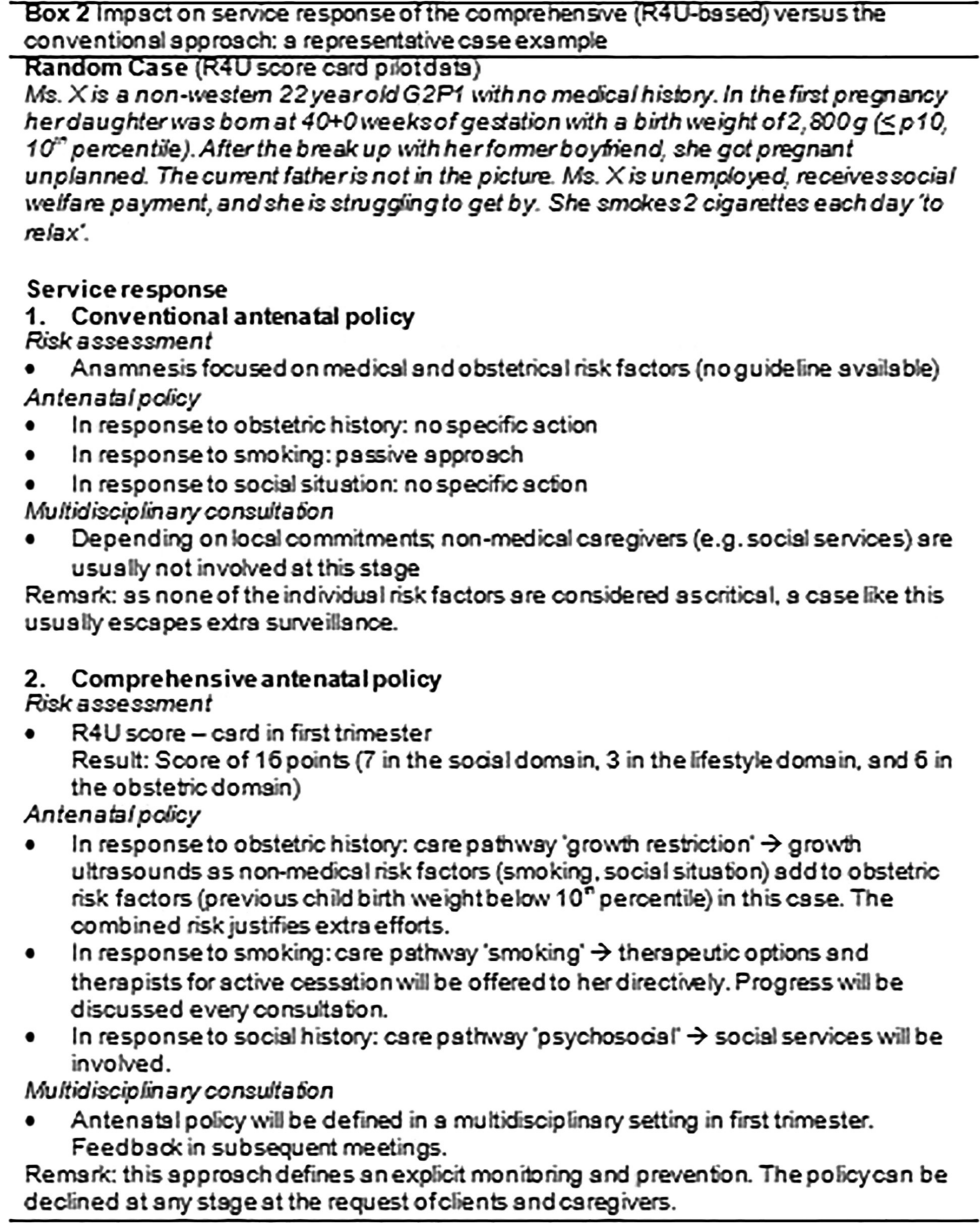


**Table 1.**
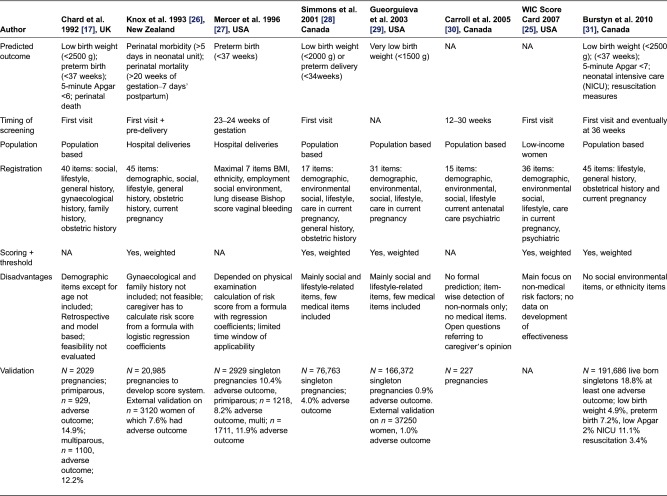

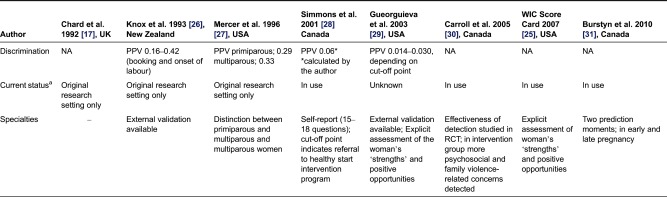
Predictive instruments for routine use in antenatal care (published from 1990 onwards)

^a^To our knowledge, NA, not available; PPV, positive predicted value; RCT, randomized controlled trial.

**Table 2.**
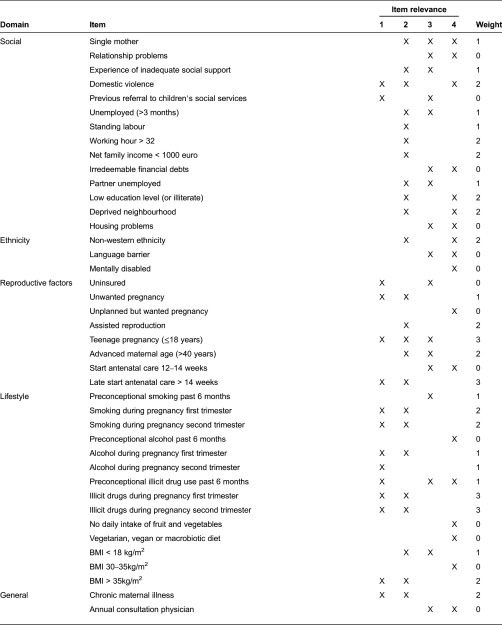

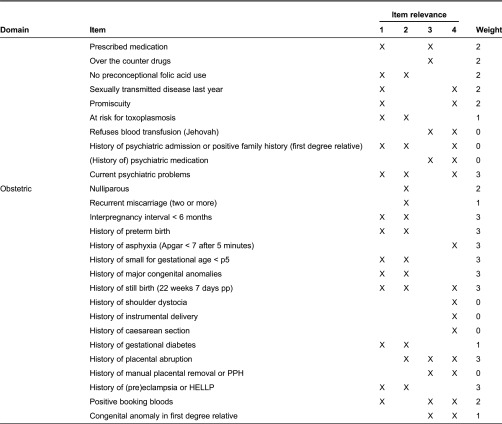
Domains and items of R4U, according to predictive and clinical management relevance

BMI, body mass index; pp, postpartum; PPH, postpartum haemorrhage; HELLP, haemolysis elevated liver enzymes and low platelets.
